# Supplementary material for: A New Parallel High-Pressure Packing System Enables Rapid Multiplexed Production of Capillary Columns
Source: Mol Cell Proteomics. 2021 Apr 20;20:100082. doi: 10.1016/j.mcpro.2021.100082 (PMC8165429; doi:10.1016/j.mcpro.2021.100082)
Supplement: Supplemental Figures S1 to S4 [file mmc3.pdf]

Supplementary Figures

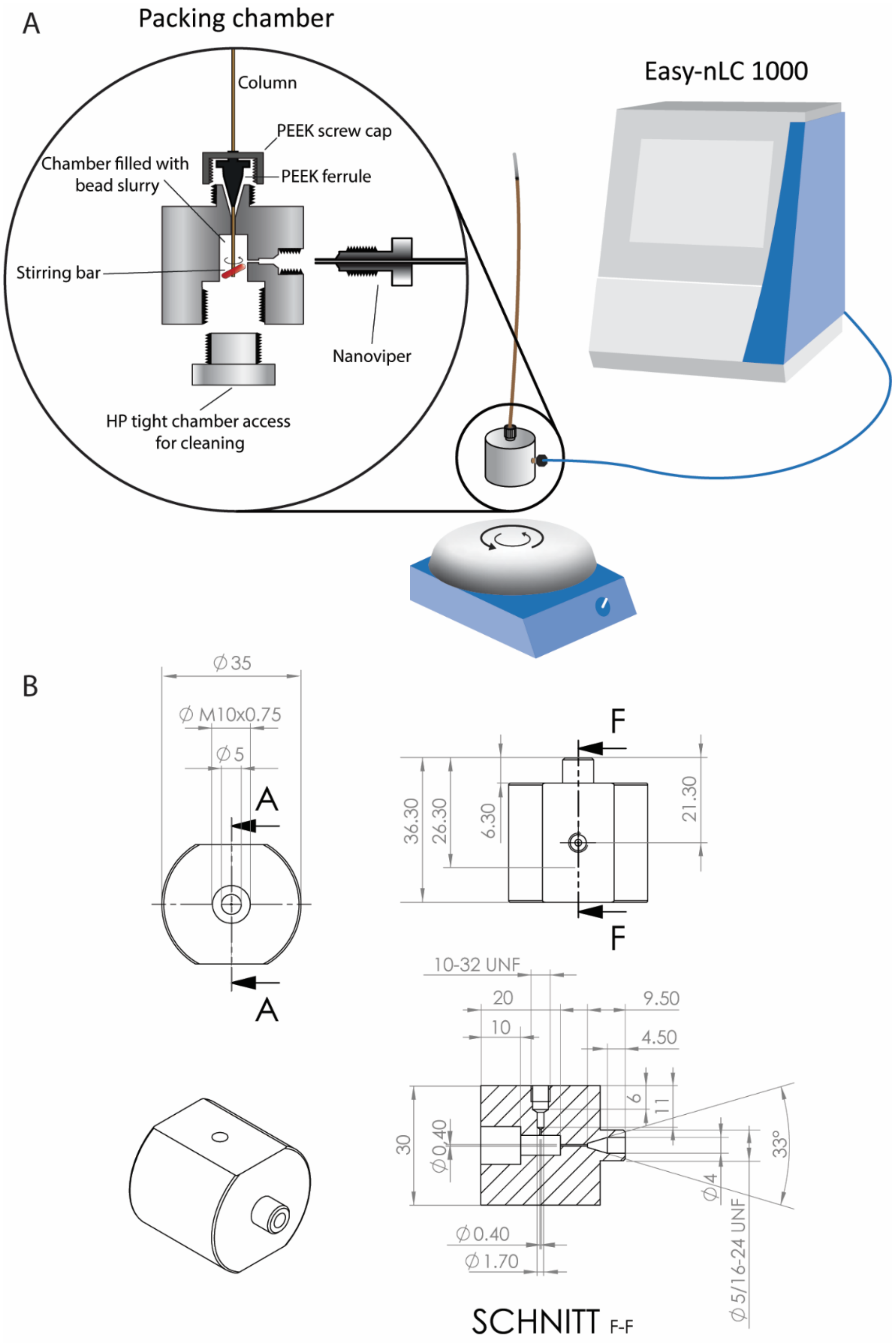

Suppl. Figure 1. Prototype one of the high-pressure packing chamber. A, An early prototype of the packing chamber for use with a LC system with nano-viper connection output. The station was used on top of a magnetic stirrer and supplied with driving fluid and pressure from an Easy-nLC 1000. **B**, Technical drawing of the standalone prototype. All distances are in mm.

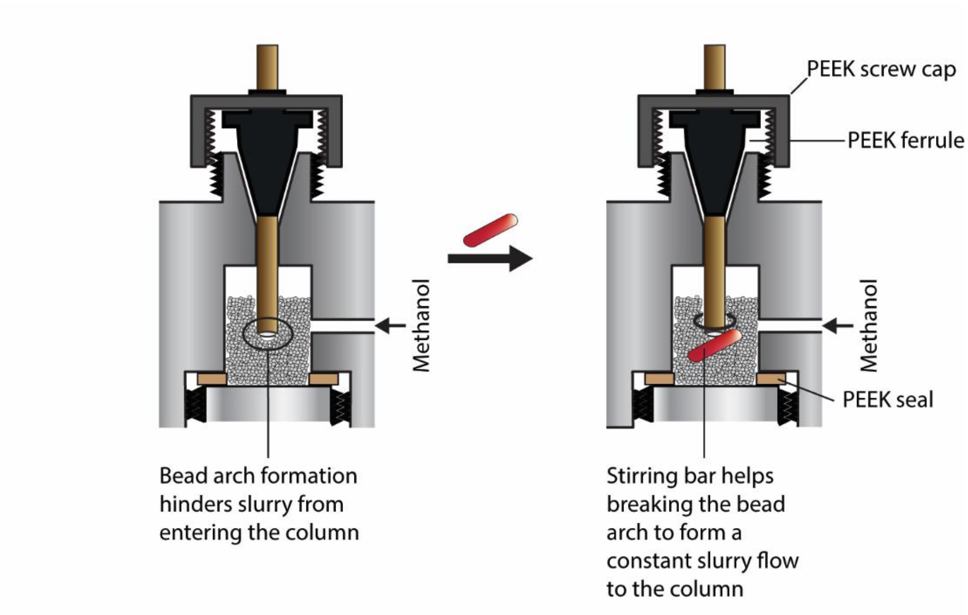

Suppl. Figure 2. Flashpack principle adapted to the high-pressure packing pot. The Flashpack principle as adapted from Kovalchuck et al. (2019) only functions, when the column entrance is in a region of high concentrated bead slurry. However, this leads to the formation of a bead arch at the column entrance and a halt of the column packing process. By consequent stirring and therefore breaking of the newly forming bead arch, a constant packing can be achieved.

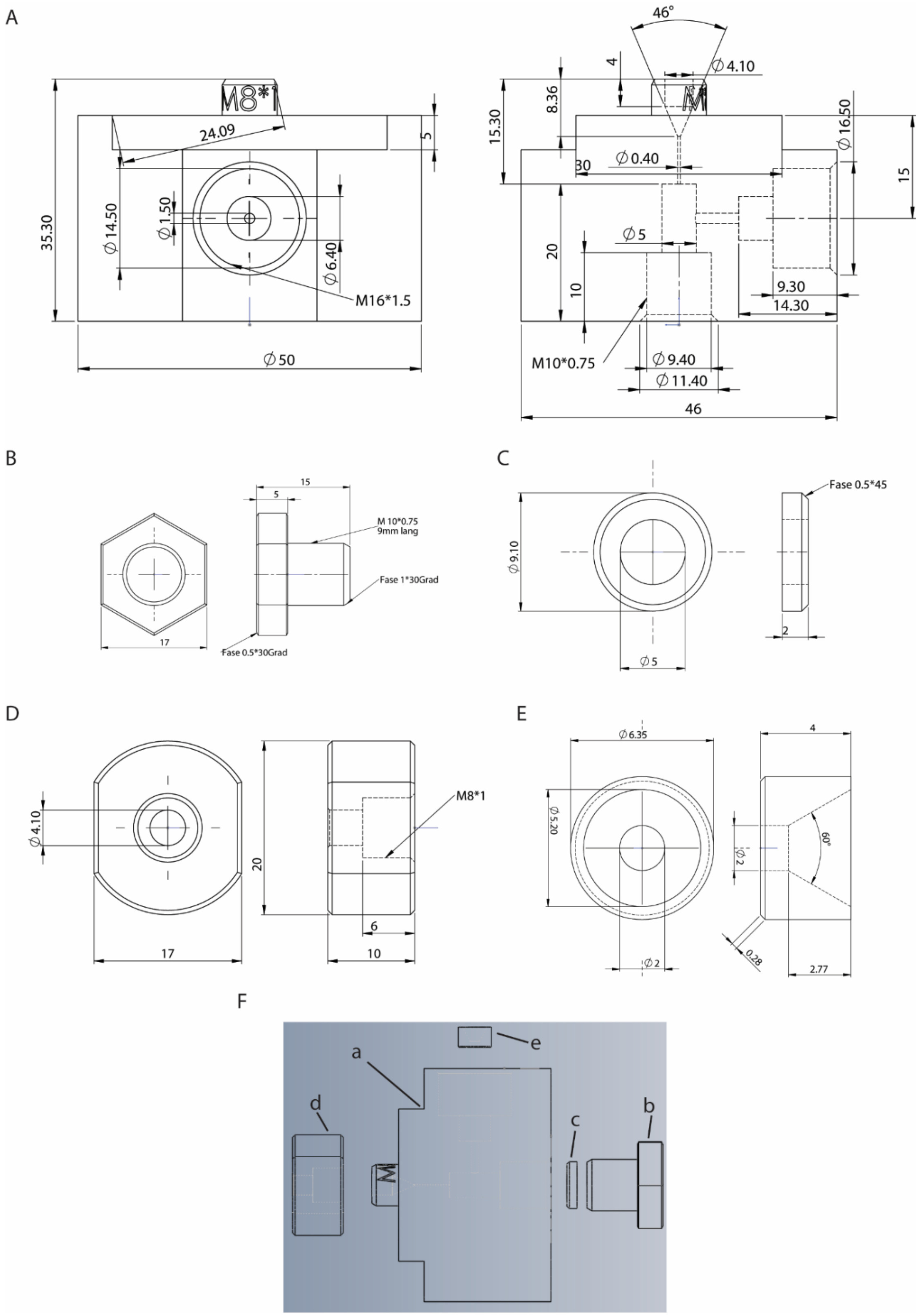

Suppl. Figure 3. Technical drawings of the packing chamber. A, Packing chamber made from stainless steel. B, Closing screw for packing chamber and prototype one (Suppl. Fig.1) made from stainless steel. C, PEEK seal ring for the closing screw. D, GF-PEEK connection screw cap to press the PEEK ferrule into the coned fitting. E, PEEK seal ring for the connection to the HP supply system. F, Assembly of the parts A-E. All distances are in mm.

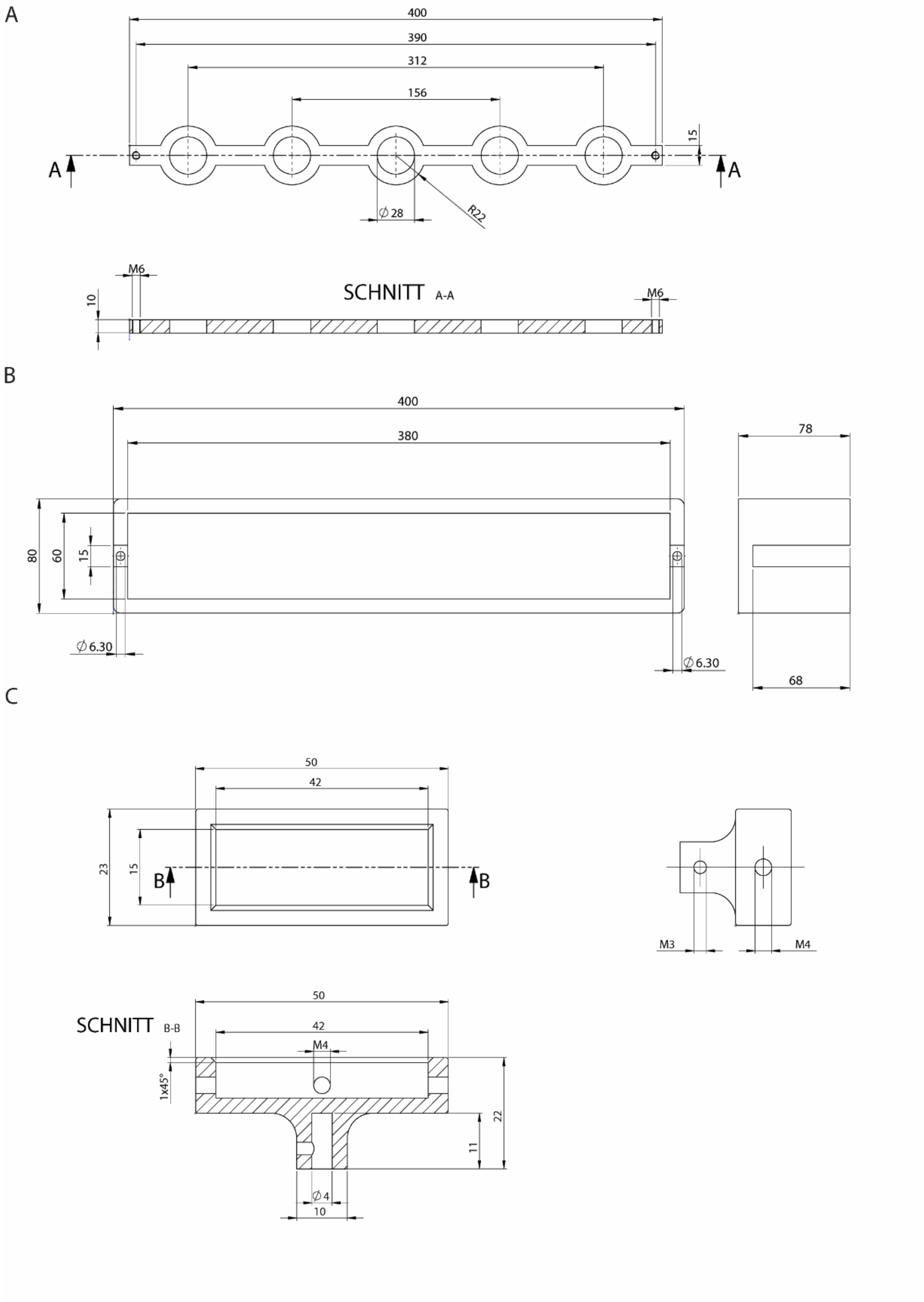

Suppl. Figure 4. Technical drawings of the stirring system. A) Frame for the electric motors. **B**) Hight adjustable stand for the magnetic holder frame in A. C) Magnet holder to be placed on the magnetic motors 3D-printed from carbon. All distances are in mm.
